# Safety and Efficacy of Transendocardial Stem Cells Therapy in Chronic Ischemic Heart Failure: A Systematic Review and Meta-analysis of Randomized Controlled Trials

**DOI:** 10.2174/011573403X353157250115105436

**Published:** 2025-01-27

**Authors:** Ibrahim Al-Sawalha, Abdel Qader Abu-Salih, Mohammad Al-Bdour, Rula Al Shimi, Mohammad Al-Slehat, Amjad Almansi, Suhel F. Batarseh, Moneeb Al-Taj, Nebras Jaloudi

**Affiliations:** 1 Department of Medicine, Princess Basma Teaching Hospital, Irbid, Jordan;; 2 Faculty of Medicine, Jordan University of Science and Technology, Irbid, Jordan;; 3 Faculty of Medicine, Al-Balqa Applied University, Al-Salt, Jordan;; 4 Department of Medicine, Prince Hamza Hospital, Amman, Jordan

**Keywords:** Stem cell therapy, chronic ischemic heart failure, mesenchymal stem cell, left ventricular function, myocardial perfusion, cardiac parameters

## Abstract

**Introduction:**

Chronic ischemic heart failure is a major global health issue despite advancements in therapy. Stem cell (SC) therapy has emerged as a potential treatment, but its effectiveness remains uncertain. This study aimed to systematically review and meta-analyze the current evidence on SC therapy's efficacy.

**Methods:**

We conducted a comprehensive literature search in PubMed, Embase, and Cochrane databases up to April 2024. We included randomized controlled trials (RCTs) with blinded designs, focusing on patients with heart failure with reduced ejection fraction (HFrEF) treated with mesenchymal stem cells compared to placebo or sham interventions *via* percutaneous endomyocardial catheter systems. Data extraction, performed independently by two authors, focused on safety and efficacy variables. The meta-analysis used a random-effects model, with sensitivity analyses to address study heterogeneity.

**Results:**

Twenty studies were included in the meta-analysis. Significant improvements were observed in the stem cell group for left ventricular end-systolic volume (LVESV) (pooled effect size -7.59, 95% CI [-12.28 to -2.89], *P=*0.002) and stress SPECT outcomes (pooled effect size -5.33, 95% CI [-6.73 to -3.93], *P*<0.00001). Sensitivity analysis reduced heterogeneity in left ventricular end-diastolic function (LVEDF) (*P=*0.01, I^2^=54%) and revealed a significant benefit for stem cell therapy (pooled effect size -3.87, 95% CI [-6.77 to -0.97], *P=*0.009). No significant effects were observed for left ventricular ejection fraction (LVEF) or myocardial oxygen consumption (MVO2). Functional improvements in New York Heart Association (NYHA) classification were noted (OR=4.22, 95% CI (1.14-15.68), *P=*0.03), though no significant differences were found in safety outcomes, including major cardiovascular events, mortality, or rehospitalization rates.

**Conclusion:**

Transendocardial SC therapy shows promise in improving certain cardiac parameters, though its impact on LVEF and MVO2 remains inconclusive, indicating the need for further research.

## 1 INTRODUCTION

Chronic ischemic heart failure with reduced ejection fraction (HFrEF) is a major cause of morbidity and mortality worldwide, in spite of huge advances in pharmacological and interventional therapies over the past few decades [1]. This condition is characterized by progressive ventricular remodeling, impaired myocardial perfusion, and a persistent decline in cardiac function, which ultimately leads to symptomatic heart failure and increased mortality [2]. Traditional therapeutic methods, including beta-blockers, angiotensin-converting enzyme inhibitors, and implantable devices, have been shown to improve outcomes. Yet, the long-term prognosis for patients with advanced ischemic heart failure remains poor [3, 4].

Despite these advances, current treatments are unable to reverse myocardial damage or halt progressive remodeling, leaving an unmet need for novel therapeutic approaches [5]. Mesenchymal stem cell (MSC) therapy has emerged as a promising alternative due to its potential to regenerate damaged myocardium, reduce fibrosis, and improve overall cardiac function [6, 7]. The transendocardial delivery of stem cells using percutaneous endomyocardial catheter systems has gained attention as a method to directly target the ischemic myocardium and improve the therapeutic efficacy of MSC interventions [8].

However, the clinical efficacy of MSC therapy in chronic ischemic heart failure remains debatable. Previous randomized controlled trials (RCTs) have shown mixed outcomes, with some demonstrating improvements in cardiac parameters such as left ventricular ejection fraction (LVEF), while others found no significant benefit [9]. These inconsistent findings have fueled ongoing debate regarding the role of MSC therapy in heart failure management and highlighted the need for a clearer understanding of its therapeutic potential [6].

In light of this crucial evidence gap, we evaluated the safety and effectiveness of transendocardial MSC treatment in patients with chronic ischemic HFrEF by conducting a comprehensive review and meta-analysis of RCTs. The main cardiac metrics that were the focus of our investigation were myocardial oxygen consumption (MVO_2_), stress single-photon emission computed tomography (SPECT), left ventricular end-systolic volume (LVESV), left ventricular end-diastolic function (LVEDF), and left ventricular EF. In order to maximize the use of MSC therapy in clinical practice, we hope to shed light on its therapeutic potential and pinpoint areas that require more investigation by synthesizing the available evidence.

## 2 MATERIALS AND METHODS

### 2.1 Search Strategy

This review is reported following the Preferred Reporting Items for Systematic Reviews and Meta-analysis (PRISMA). We used the PICOS (participants, intervention, comparison, and outcomes of study design) model to design our study question. We conducted an extensive and comprehensive search of randomized control trials (RCTs) in five major databases (PubMed, ScienceDirect, Scopus, EBSCOhost, and Cochrane Library) up to April 2024 regarding the effects of stem cell therapy on myocardial function in patients suffering from ischemic heart failure with reduced ejection fraction. All searches were conducted and saved in EndNote version 20 for Windows.

### 2.2 Search Terms

The search strategy employed a combination of keywords and Medical Subject Headings(MeSH) terms: (stem cell[Title/Abstract] OR cell therapy[Title/Abstract] OR progenitor[Title/Abstract] OR mesenchymal[Title/Abstract] OR “bone marrow mononuclear”[Title/Abstract] OR myoblast[Title/Abstract] OR autologous[Title/Abstract] OR allogenic[Title/Abstract]) AND (heart failure[Title/Abstract] OR cardiomyopathy[Title/Abstract] OR myocardium[Title/Abstract] OR myocardial[Title/Abstract] OR coronary[Title/Abstract]) AND (endocardial OR transendocardial OR intramuscular OR intramyocardial OR endomyocardial) AND (randomized OR placebo OR control)* NOT (animal OR mice OR rat OR rats OR rodent OR sheep OR pork OR murine). The search terms were refined using Boolean operators (“AND,” “OR”) as per the Cochrane Handbook for Systematic Reviews of Interventions (Chapter 4.4.4). Additional studies were identified through manual reference searches. In addition, we checked the references listed in the studies to make sure that we were reviewing all available information. Duplicate records were deleted after titles and abstracts were screened using EndNote TM. Studies were screened by two independent authors and articles that satisfied our inclusion criteria were sought for in full-text articles. Discrepancies among the authors were resolved through detailed discussions.

### 2.3 Inclusion and Exclusion Criteria

Our eligibility criteria for study selection were: 1) Age 18 and above, 2) randomized controlled trials (RCTs) with a double-blind or single-blind design, 3) patients diagnosed with chronic ischemic heart failure with reduced ejection fraction (HFrEF) <45%, 4) utilizing mesenchymal stromal cells as the intervention and comparing its efficacy with placebo or sham controls, 5) treatment either with 3D electromechanical guided (NOGA) or other percutaneous endomyocardial (synonymous intra-myocardial or trans-endocardial) catheter systems, 6) trials reporting primary and/or secondary outcomes related to cardiac function and mortality rates, including all-cause mortality, and 7) studies published in English. Articles were excluded if one of the following were present: 1) patients with heart failure due to etiologies other than chronic ischemic heart disease (*e.g.*, congenital heart disease, non-ischemic cardiomyopathy), 2) interventions like gene therapy, intracoronary delivery, venous delivery, or surgical direct intra-myocardial injections, 3) non-randomized clinical trials, case reports, case series, reviews, and meta-analyses studies, 4) acute coronary syndrome within 6 weeks of inclusion, 5) studies reporting outcomes not relevant to cardiac function or mortality, 6) studies published in languages other than English, and 7) studies with incomplete data or a high risk of bias.

### 2.4 Data Extraction

Data extraction was carried out by two independent investigators using a standardized Google Spreadsheet, who thoroughly reviewed the full articles and extracted data from eligible ones. Extracted information included patient demographics such as age, gender, baseline left ventricular ejection fraction (LVEF), comorbidities (*e.g.*, diabetes, hypertension, hyperlipidemia), and previous cardiac interventions (*e.g.*, CABG, PCI). Details about the intervention were also recorded, including the type and dose of mesenchymal stromal cells (MSCs), their source (autologous or allogenic), the delivery method (*e.g.*, NOGA or other catheter-based systems), and the frequency of administration. Outcomes included changes in LVEF as the primary endpoint, along with secondary outcomes such as left ventricular end-systolic volume (LVESV), left ventricular end-diastolic volume (LVEDV), New York Heart Association (NYHA) classification, hospitalizations due to decompensated HF, all-cause mortality, and adverse events. Imaging modalities used to assess outcomes (*e.g.*, SPET CT, echocardiography, or MRI) were also extracted, along with the follow-up duration and any procedural complications reported. The data were then cross-checked for accuracy by two additional authors.

### 2.5 Assessment of Risk of Bias

The risk of bias for each included study was independently evaluated by two reviewers using the Cochrane Collaboration’s Risk of Bias Tool. This tool assesses seven domains: random sequence generation, allocation concealment, blinding of participants and personnel, blinding of outcome assessment, incomplete outcome data, selective reporting, and other biases. Each domain was classified as low, high, or unclear risk of bias based on the information provided in the study reports. Discrepancies between the reviewers were resolved through discussion, and a third reviewer was consulted when necessary. The overall risk of bias for each study was determined and included in the synthesis of evidence to ensure that the findings are robust and reliable.

### 2.6 Statistical Analysis

Meta-analysis was conducted using Review Manager 5.4. It was performed using the random-effects model to account for potential variations among studies. We calculated pooled effect sizes for the primary outcomes, including changes in left ventricular end-systolic volume (LVESV), stress SPECT, left ventricular end-diastolic function (LVEDF), left ventricular ejection fraction (LVEF), and myocardial oxygen consumption (MVO_2_). The effect sizes were reported as mean differences with corresponding 95% confidence intervals (CIs). Heterogeneity among the included studies was assessed using the I^2^ statistic, with an I^2^ value greater than 50% indicating substantial heterogeneity. In cases where significant heterogeneity was detected, a sensitivity analysis was conducted by sequentially excluding one study at a time to identify potential sources of heterogeneity and assess the robustness of the results. Statistical significance was determined at a *p*-value of less than 0.05. All analyses were performed using RevMan software (version 5.4), ensuring rigorous data handling and interpretation [10-28].

## 3 RESULTS

### 3.1 Characteristics of Included Studies

**Fig. (1):**
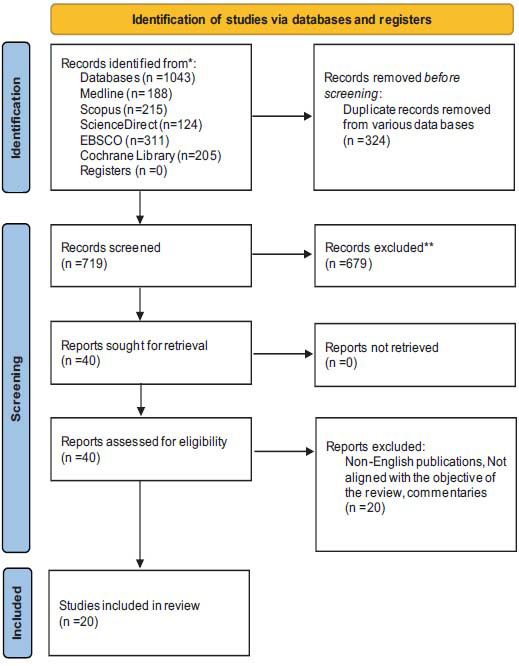
PRISMA flow diagram illustrating the selection process and the number of studies identified, screened, excluded, and included at each stage.

From an initial pool of 1,043 citations identified through database searches, 20 studies were included in the final meta-analysis. The databases searched included Medline (188 citations), Scopus (215), ScienceDirect (124), EBSCO (311), and the Cochrane Library (205). After the removal of duplicate records (n = 324), 719 unique studies were screened for relevance. Of these, 679 were excluded for not meeting the eligibility criteria, and 40 full-text reports were assessed for retrieval. Twenty studies were excluded for reasons such as being non-English, irrelevant to the research objective, or commentary articles. Ultimately, 20 studies were selected for inclusion in the review (Fig. **1**).

The included studies were all published within the last 15 years, with most conducted in Europe and North America. They predominantly consisted of randomized controlled trials (RCTs), and the majority of participants were male. The studies were assessed for quality, revealing a low risk of bias across most domains, thereby ensuring the reliability of findings in this systematic review and meta-analysis. A detailed summary of the characteristics of the included studies is provided in Table **1**, while an assessment of the risk of bias is presented in Fig. (**2**).

### 3.2 Efficacy Outcomes

**Fig. (4):**
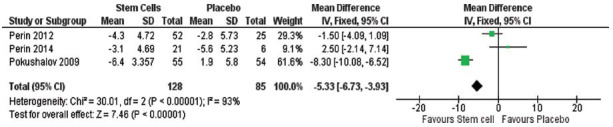
The overall Change in Stress SPECT between the stem cells and the Placebo favored the stem cells (pooled effect size -5.33, 95% CI (-6.73 to -3.93), *P=*0.00001). Pooled studies are not homogenous (Chi-square *P=*0.000001, I^2^ =93%).

The meta-analysis focused on multiple efficacy outcomes, including changes in left ventricular end-systolic volume (LVESV) and stress single-photon emission computed tomography (SPECT). A significant reduction in LVESV was observed in the stem cell group compared to the control group, with a pooled effect size of -7.59 (95% CI [-12.28 to -2.89], *p =* 0.002), as shown in Fig. (**3**). Similarly, stress SPECT outcomes also significantly favored the stem cell group, with a pooled effect size of -5.33 (95% CI (-6.73 to -3.93), *P* < 0.00001; Fig. **4**).

**Fig. (5):**
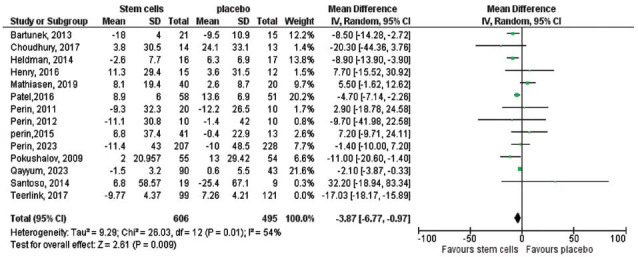
Sensitivity analysis of the overall change in LVEDF between the Stem Cells and Placebo groups. The initial pooled effect size was -4.66 (95% CI (-10.39 to 1.06), *P=*0.11), indicating no significant difference. Upon excluding the study by Teerlink *et al.* (2017), heterogeneity was reduced (*P=*0.01, I^2^=54%), and the pooled effect size became significant, favoring the Stem Cells group (effect size -3.87, 95% CI (-6.77 to -0.97), *P=*0.009).

**Fig. (6):**
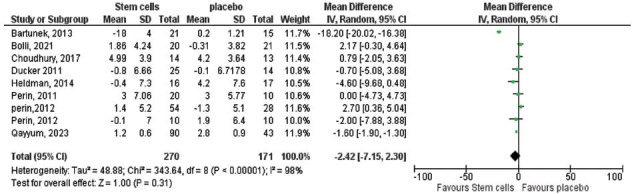
The overall change in LVEF between the stem cells and the placebo groups did not favor either of the two groups (pooled effect size 0.08, 95% CI (-0.1 to 0.26), *P=*0.39). Pooled studies not homogenous (Chi-square *P=*0.000001, I^2^ =98%).

**Fig. (7):**
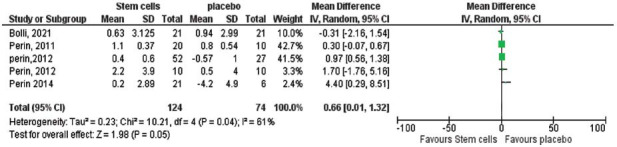
The overall change in MVO_2_ between the stem cells and placebo groups did not favor either of the two groups (pooled effect size 0.66, 95% CI (0..1 to 1.32), *p=*0.05). Pooled studies are not homogenous (Chi-square *P=*0.04, I^2^=61%).

Initially, there was no significant difference in left ventricular end-diastolic function (LVEDF) between the two groups. However, sensitivity analyses excluding one study at a time reduced heterogeneity (*P =* 0.01, I^2^ = 54%) and revealed a significant improvement in the stem cell group, with a pooled effect size of -3.87 (95% CI (-6.77 to -0.97), *P =* 0.009; Fig. **5**). On the other hand, changes in left ventricular ejection fraction (LVEF) and myocardial oxygen consumption (MVO2) did not show statistically significant differences between the groups. For LVEF, the pooled effect size was 0.08 (95% CI (-0.10 to 0.26), *P =* 0.39; Fig. **6**), while for MVO_2_, the pooled effect size was 0.66 (95% CI (-0.10 to 1.32), *P =* 0.05; Fig. **7**).

### 3.3 Safety Outcomes

Safety outcomes were assessed across all included studies. The overall rate of complications did not differ significantly between the stem cell and control groups, with a pooled effect size of 1.22 (95% CI (0.78 to 1.90), *P =* 0.37). Similarly, no significant differences were observed in the rates of major cardiovascular events, such as stroke, between the two groups (pooled effect size: 1.05, 95% CI (0.55 to 2.01), *P =* 0.89).

Specific adverse events, including arrhythmias and myocardial infarctions, were reported in individual studies but did not significantly influence the overall safety profile. For rehospitalization outcomes, no significant difference was found between the groups (OR = 0.85, 95% CI: 0.55–1.31, *P =* 0.45). This analysis included 357 participants in the stem cell group and 226 in the placebo group, with low heterogeneity (I^2^ = 0%, *P =* 0.92), indicating consistency among the studies. Similarly, for mortality outcomes, the meta-analysis revealed a non-significant trend towards higher mortality in the stem cell group (effect size = 1.67, *P =* 0.09), with low heterogeneity (I^2^ = 13%). In terms of functional outcomes, stem cell therapy showed a significant improvement in New York Heart Association (NYHA) classification (OR = 4.22, 95% CI: 1.14-15.68, *P =* 0.03). Furthermore, there was no significant difference in the odds of NYHA worsening between the two groups (OR = 0.42, 95% CI: 0.12–1.52, *p =* 0.19), with moderate heterogeneity (I^2^ = 46%). A pictorial representation of our main results is presented in Fig. (**8**) [6, 29-42].

## 4 DISCUSSION

In our meta-analysis assessing the influence of MSC therapy on outcomes in heart failure patients, the change in left ventricular end-systolic volume (LVESV) and the results of stress SPECT demonstrated a significant advantage for the stem cell group. However, the overall changes in left ventricular ejection fraction (LVEF) and myocardial oxygen consumption (MVO_2_) showed no advantage for either group. Regarding safety outcomes assessment, the overall complication rates were similar between the stem cell and control groups, with no significant differences in stroke or major cardiovascular events. Although arrhythmias and myocardial infarctions were reported in several studies, they did not significantly affect the overall safety profile.

Cell-based therapy for ischemic and nonischemic heart disease utilizes various sources and cell types, such as autologous or allogeneic bone marrow cells and MSCs, delivered through intramyocardial injections, intracoronary infusion, or occasionally intravenous infusion [43-45].

Embryonic stem cells (ESCs) originate from the inner cell mass (ICM) of the blastocyst and possess two key features: the ability to undergo indefinite proliferation, known as “self-renewal”, and the capacity to differentiate into various cell types, referred to as “pluripotency” [46]. These unique properties are tightly regulated by complex external and internal mechanisms, including transcription factors, epigenetic and histone modifications, and signaling pathways [46].

Stem cell therapy for heart diseases involves various cell types, each with unique properties and potential [47]. Bone marrow mononuclear cells (BMMNCs) and mesenchymal stem cells (MSCs) are the most extensively studied in ischemic heart disease [48-51]. BMMNCs are a mixed population of progenitor cells with modest regenerative effects, while MSCs, known for their anti-inflammatory and regenerative properties, may provide greater improvement in cardiac function, particularly left ventricular ejection fraction (LVEF) [47, 50]. The choice of cell type is crucial due to functional differences that influence therapeutic outcomes [47].

Stem cell therapy for heart diseases employs two primary types: autologous (auto) and allogeneic (allo). Autologous therapy utilizes the patient’s own cells, reducing risks of immune rejection, while allogeneic therapy uses donor cells, offering advantages such as availability, scalability, and disease-free cell products [20, 43, 52]. Emerging evidence suggests that allo-hMSCs may have superior therapeutic efficacy over auto-hMSCs in conditions like nonischemic dilated cardiomyopathy (NIDCM), owing to their strong anti-inflammatory and regenerative properties [43]. These findings support the potential for pivotal trials to establish allo-hMSC as a standard treatment option.

The therapeutic effectiveness of MSC therapy in heart failure patients may arise from several mechanisms, including promoting cell differentiation, reducing fibrosis, new blood vessel formation, modulating inflammation, and lowering myocardial cell death [16]. The preservation of MSCs is a key factor in their effectiveness [10]. An *in vitro* study demonstrated that cryopreserved MSCs maintained their characteristic marker expression and exhibited comparable proliferation capabilities [17]. Likewise, the MSC delivery method is crucial for heart failure outcomes. Intracoronary injection was observed to reduce mortality and improve the 6MWT more efficiently than transendocardial stem cell injection (TESI) [10]. However, Chin *et al.* [15] found both methods improved LV function and remodeling, with TESI trending towards greater benefits, while preclinical studies by Perin *et al.* [18] and Rigol *et al.* [19] showed varying advantages for TESI and intracoronary injection, respectively. Differences in outcomes may be due to factors like number of homing cells and their local microenvironment [20].

Cell-based therapy offers a promising and safe option for addressing unmet needs in heart disease treatment [6]. Despite numerous phase I and II clinical trials, there is ongoing controversy as to the ideal patient population for cell treatment [42]. While ischemic cardiomyopathy (ICM) and dilated cardiomyopathy (DCM) differ in pathology and prognosis, mesenchymal stem cell (MSC) therapy has shown benefits for both [41, 42]. In chronic ischemia, human MSCs help counteract cardiac remodeling, while in DCM, they primarily enhance functional aspects of heart failure. However, studies suggest MSC therapy may be more effective in DCM due to its progressive nature and severe prognosis, though the ideal patient population remains debated [42].

The significant reduction in left ventricular end-systolic volume (LVESV) in the stem cell group is consistent with previous studies suggesting that stem cell therapy can contribute to left ventricular remodeling by reducing systolic wall stress and preventing further dilation of the heart [48], while the improvement in SPECT may indicate enhanced angiogenesis and myocardial repair, leading to better oxygenation and contractility [49].

The initial non-significant findings for left ventricular end-diastolic function (LVEDF) may stem from variations in study design and patient populations. However, the sensitivity analysis that showed significant improvement with reduced heterogeneity reinforces the potential of stem cell therapy to improve diastolic function. Similar trends have been noted in a previous meta-analysis, which found a trend toward diastolic function improvement, especially in patients treated earlier in the disease course [48].

Conversely, our findings did not show a significant effect on left ventricular ejection fraction (LVEF), in line with the mixed results observed in previous reviews. While some analyses have reported improvements in LVEF [12, 50], others suggest that LVEF might not fully capture the benefits of stem cell therapy, as remodeling and perfusion improvements often occur independently of changes in ejection fraction [43]. Similarly, myocardial oxygen consumption (MVO_2_) showed no significant improvement, which might reflect the need for long-term follow-up to detect metabolic changes.

Other studies have highlighted additional efficacy outcomes of mesenchymal stem cell (MSC) therapy in heart failure. Both Shen *et al.* and Fan *et al.* observed trends toward reduced mortality and rehospitalization rates among MSC-treated patients [10, 48]. These studies also reported improvements in functional capacity, as measured by the six-minute walk test, and reductions in heart failure severity based on NYHA classification. These findings highlight the promise of MSC therapy in improving diverse clinical outcomes but emphasize the need for standardized evaluation methods to confirm its effectiveness.

In our meta-analysis, the overall complication rate and specific adverse events, including stroke and MI, were not significantly different between MSC-treated and control groups. Our findings align with previous studies that reported no statistically significant differences in the incidence of myocardial infarction (MI), heart failure recurrence, or mortality between mesenchymal stem cell (MSC) therapy and control groups [11, 35]. Similarly, Lalu *et al.* found no significant association between MSC administration and overall acute adverse events (AEs) such as arrhythmias, MI, or pericardial effusion, though they noted an increased risk for delayed neurological AEs with MSC therapy [13]. These consistent findings across studies reinforce the safety profile of MSC therapy, suggesting it does not increase the risk of major complications while still offering potential efficacy benefits.

Overall, transendocardial stem cell therapy has shown encouraging results, notably improving certain cardiac parameters. Moreover, a comparable safety profile to the control group has been demonstrated, with no significant differences in complication rates, including stroke and major cardiovascular events. However, despite its potential, the effects on key measures such as LVEF and MVO_2_ remain uncertain, especially after sensitivity adjustments. These outcomes underscore the necessity for further research to fully explore the potential of stem cell treatments in managing heart failure.

## CONCLUSION

In conclusion, our meta-analysis underscores the clinical potential of transendocardial stem cell therapy in managing chronic ischemic heart failure. The therapy demonstrated significant benefits in improving key cardiac parameters, particularly left ventricular end-systolic volume (LVESV) and stress SPECT outcomes, which are critical indicators of ventricular remodeling and perfusion. However, its lack of significant impact on left ventricular ejection fraction (LVEF) and myocardial oxygen consumption (MVO_2_) highlights the need for further refinement in patient selection and therapeutic protocols. Importantly, the therapy exhibited a favorable safety profile, with no increased risk of stroke or major cardiovascular events compared to controls. These findings highlight the potential of stem cell therapy as a treatment modality in heart failure, though further research is essential to clarify its effects on critical clinical outcomes and optimize therapeutic strategies.

## Figures and Tables

**Fig. (2):**
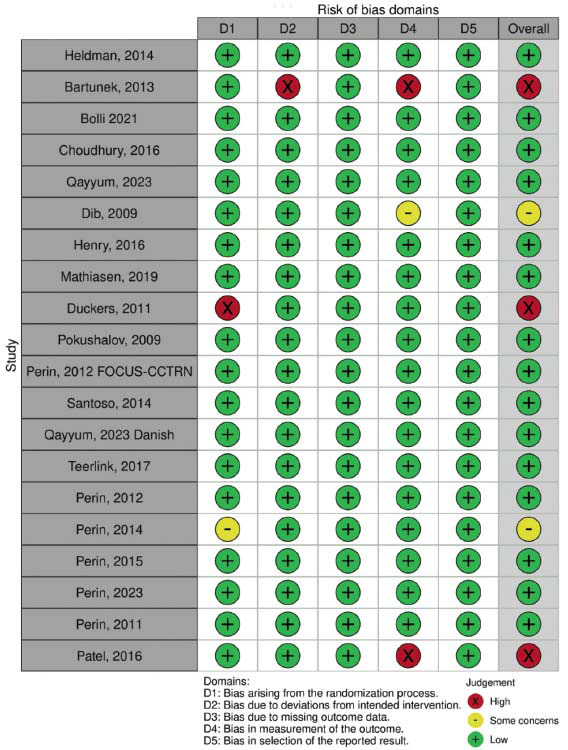
Risk of bias assessment for included studies using the Cochrane risk-of-bias tool. The figure displays the proportion of studies with low, unclear, and high risk of bias across different domains, highlighting the overall quality and reliability of the evidence.

**Fig. (3):**
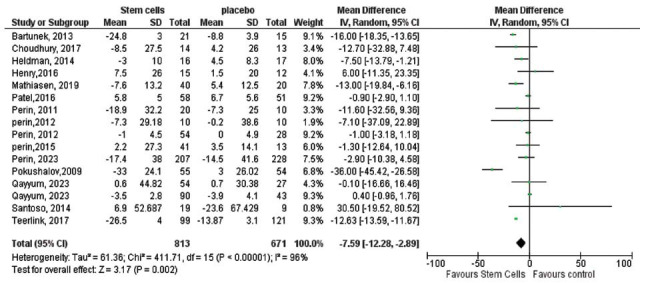
The overall change in LVESV mL was statistically significant. Pooled effect size was significant -7.59, 95% CI (-12.28 to-2.89), *P=*0.002. Pooled studies are not homogenous (Chi-square *P=*0.000001, I-square=93%).

**Fig. (8):**
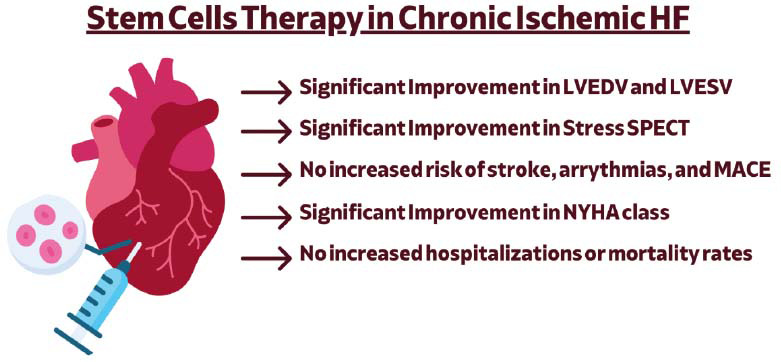
A pictorial representation of our main results. **Abbreviations:** LVEDV; Left ventricular end-diastolic volume, LVESV; Left ventricular end-systolic volume, SPECT; Single-Photon emission computed tomography, NYHA; New York Heart Association, MACE; Major adverse cardiovascular events.

**Table 1:** Characteristics of the included studies.

**Author, Year**	**Intervention (n)**	**Intervention: Mean Age (Years) (SD)**	**Placebo (n)**	**Placebo: Mean Age (Years) (SD)**	**Cells (Type)**	**Allogenic/ Autogenic**	**NYHA**	**Imaging Modality to Assess LV Function**	**Follow Up Period**
Heldman *et al.,* 2014 [21]	BMC: 19, SC: 19	SC =57.1 (±10.6), BMC =61.1 (±8.4)	SC placebo: 10, BMC placebo: 10	SC placebo: 60 (±12), BMC placebo: 61.3 (±9)	Mesenchymal stem cell, mononuclear bone marrow cells	Autologus	I-III	Cardiac MRI with gadolinium contrast, contrast-enhanced CT, and echocardiography	12 months
Bartunek *et al.,* 2013 [22]	21	55.7 (±10.4)	15	59.5 (±8)	Cardiopoietic mesenchymal stem cells	Autologus	II-III	Echocardiography	24 months
Bolli *et al.,* 2021 [23]	MSCs+CPCs =33, MSCs =29, CPCs =11	MSCs+CPCs= 61 (±11.1), MSCs= 61.7 (±6.7), CPCs= 64.2 (±8.1)	32	63.1 (±8.8)	Bone marrow derived mesenchymal stromal cells (MSCs), c-kit positive cardiac cells (CPCs)	Autologus	II-III	MRI	12 months
Choudhury *et al.,* 2016 [24]	15	65.3 (±9.4)	15	60.4 (±11.2)	Bone marrow-derived cells	Autologus	II-IV	Cardiac magnetic resonance imaging, or cardiac CT for those unable to undergo CMRI	12 months
Qayyum *et al.,* 2023 [25]	90	66.4 (±8.1)	43	64 (±8.8)	Adipose tissue derived mesenchymal stromal cells	Allogenic	II-III	Echocardiography	6 months
Dib *et al.,* 2009 [26]	12	65.1	11	62	Skeletal myoblasts	Autologus	II-IV	Echocardiography	12 months
Henry *et al.,* 2016 [27]	17	64.1 (±8.2)	14	65.7 (±7.3)	Adipose-derived regenerative cells	Autologus	II-III	Echocardiography	12 months
Mathiasen *et al.,* 2019 [28]	40	66.1 (±7.7)	20	64.2 (±10.6)	Bone marrow-derived mesenchymal stromal cell	Autologus	II-III	MRI or CT for those unable to undergo MRI	6 months
Duckers *et al.,* 2011 [29]	26	59.2 (±8.64)	14	61.9 (±8.38)	Skeletal myoblasts	Autologus	II-III	MUGA	6 months
Pokushalov *et al.,* 2009 [30]	55	61 (±9)	54	62 (±5)	Bone marrow mononuclear cells	Autologus	N/A	Echocardiography, SPECT	12 months
Perin *et al.,* 2012 [31]	61	63.95 (±10.9)	31	62.32 (±8.25)	Bone marrow mononuclear cells	Autogenic	II-III	Echocardiography, SPECT, and MRI	6 months
Santoso *et al.,* 2014 [32]	19	58 (±9.9)	9	60 (±5.6)	Bone marrow mononuclear cells	Autogenic	III-IV	Echocardiography, SPECT, and MRI	6 months
Qayyum *et al.,* 2023 [33]	54	67 (±9)	27	66.6 (±8.1)	Adipose tissue derived mesenchymal stromal cells	Allogenic	II-III	Echocardiography	6 months
Teerlink *et al.,* 2017 [34]	120	61.6 (±8.59)	151	62.1 (±8.72)	Cardiopoietic mesenchymal stem cells	Autogenic	II-IV	Echocardiography	12 months
Perin *et al.,* 2012 [35]	10	58.2 (±6.1)	10	57.8 (±5.5)	Aldehyde dehydrogenase–bright stem cells	Autogenic	II-III	Echocardiography, and SPECT	6 months
Perin *et al.,* 2014 [36]	21	65.8 (±6.3)	6	55.7 (±6.1)	Adipose-derived regenerative cells	Autogenic	II-III	Echocardiography, SPECT, and MRI	18-36 months
Perin *et al.,* 2015 [37]	45	62.2 (±10.3)	15	62.7 (±11.2)	Mesenchymal precursor cells	Allogenic	II-III	Echocardiography, SPECT, and MUGA Scan	36 months
Perin *et al.,* 2023 [38]	283	62.7 (±10.9)	282	62.6 (±10.4)	Mesenchymal precursor cells	Allogenic	II-III	Echocardiography, and MUGA Scan	12 months
Perin *et al.,* 2011 [39]	20	60.5 (±6.4)	10	56.3 (±8.6)	Bone marrow mononuclear cells	Autogenic	III-IV	Echocardiography, and SPECT	6 months
Patel *et al.,* 2016 [40]	58	65.3 (±8.49)	51	64.7 (±9.94)	Ixmyelocel-T cell	N/A	III-IV	Echocardiography	12 months

**Abbreviations:** SC: Stem cells, MSCs: Mesenchymal stem cells, BMC: Bone marrow concentrate, CPC: Cardiac progenitor cell, MUGA: multigated acquisition, CMRI: Cardiac magnetic resonance imaging, MRI: Magnetic resonance imaging, CT: Computed tomography, SPECT: Single-photon emission computed tomography.

## Data Availability

All data related to this manuscript can be shared upon reasonable request from the corresponding author.

## LIST OF ABBREVIATIONS

SC: Stem Cell
RCTs: Randomized Controlled Trials
HFrEF: Heart Failure with Reduced Ejection Fraction
LVEDF: Left Ventricular End-diastolic Function
LVEF: Left Ventricular Ejection Fraction
NYHA: New York Heart Association
MVO_2_: Myocardial Oxygen Consumption
PICOS: Participants, Intervention, Comparison, and Outcomes of Study Design
BMMNCs: Bone marrow mononuclear cells
MSCs: Mesenchymal Stem Cells
NIDCM: Nonischemic Dilated Cardiomyopathy

## SUPPLEMENTARY MATERIAL

PRISMA checklist is available as supplementary material on the publisher’s website along with the published article.

## References

[1] McMurray J.J.V., Adamopoulos S., Anker S.D. (2012). ESC Guidelines for the diagnosis and treatment of acute and chronic heart failure 2012: The Task Force for the Diagnosis and Treatment of Acute and Chronic Heart Failure 2012 of the European Society of Cardiology. Developed in collaboration with the Heart Failure Association (HFA) of the ESC.. Eur. Heart J..

[2] Benjamin E.J., Muntner P., Alonso A. (2019). Heart disease and stroke statistics-2019 update: A report from the American Heart Association.. Circulation.

[3] Ziaeian B., Fonarow G.C. (2016). Epidemiology and aetiology of heart failure.. Nat. Rev. Cardiol..

[4] Ponikowski P., Voors A.A., Anker S.D. (2016). 2016 ESC Guidelines for the diagnosis and treatment of acute and chronic heart failure.. Eur. Heart J..

[5] Sapna F.N.U., Raveena F.N.U., Chandio M. (2023). Advancements in heart failure management: A comprehensive narrative review of emerging therapies.. Cureus.

[6] Karantalis V., Hare J.M. (2015). Use of mesenchymal stem cells for therapy of cardiac disease.. Circ. Res..

[7] Kastrup J., Mygind N.D., Qayyum A.A., Mathiasen A.B., Haack-Sørensen M., Ekblond A. (2016). Mesenchymal stromal cell therapy in ischemic heart disease.. Scand. Cardiovasc. J..

[8] Gyöngyösi M., Wojakowski W., Lemarchand P. (2015). Meta-analysis of cell-based cardiac studies (ACCRUE) in patients with acute myocardial infarction based on individual patient data.. Circ. Res..

[9] Fisher S.A., Doree C., Mathur A., Taggart D.P., Martin-Rendon E. (2016). Stem cell therapy for chronic ischaemic heart disease and congestive heart failure.. Cochrane Libr..

[10] Fan M., Huang Y., Chen Z. (2019). Efficacy of mesenchymal stem cell therapy in systolic heart failure: A systematic review and meta-analysis.. Stem Cell Res. Ther..

[11] Fu H., Chen Q. (2020). Mesenchymal stem cell therapy for heart failure: A meta-analysis.. Herz.

[12] Jayaraj J.S., Janapala R.N., Qaseem A. (2019). Efficacy and safety of stem cell therapy in advanced heart failure patients: A systematic review with a meta-analysis of recent trials between 2017 and 2019.. Cureus.

[13] Lalu M.M., Mazzarello S., Zlepnig J. (2018). Safety and efficacy of adult stem cell therapy for acute myocardial infarction and ischemic heart failure (safecell heart): A systematic review and meta-analysis.. Stem Cells Transl. Med..

[14] Hare J.M., Traverse J.H., Henry T.D. (2009). A randomized, double-blind, placebo-controlled, dose-escalation study of intravenous adult human mesenchymal stem cells (prochymal) after acute myocardial infarction.. J. Am. Coll. Cardiol..

[15] Chen S., Fang W., Ye F. (2004). Effect on left ventricular function of intracoronary transplantation of autologous bone marrow mesenchymal stem cell in patients with acute myocardial infarction.. Am. J. Cardiol..

[16] Krishna Mohan G.V., Tirumandyam G., Vemulapalli H.S., Vajje J., Asif H., Saleem F. (2023). Mesenchymal stem cell therapy for a better prognosis of heart failure: A systematic review and meta-analysis of randomized controlled trials.. Cureus.

[17] Haack-Sorensen M., Bindslev L., Mortensen S., Friis T., Kastrup J. (2007). The influence of freezing and storage on the characteristics and functions of human mesenchymal stromal cells isolated for clinical use.. Cytotherapy.

[18] Perin E.C., Silva G.V., Assad J.A.R. (2008). Comparison of intracoronary and transendocardial delivery of allogeneic mesenchymal cells in a canine model of acute myocardial infarction.. J. Mol. Cell. Cardiol..

[19] Rigol M., Solanes N., Farré J. (2010). Effects of adipose tissue-derived stem cell therapy after myocardial infarction: Impact of the route of administration.. J. Card. Fail..

[20] Golpanian S., Wolf A., Hatzistergos K.E., Hare J.M. (2016). Rebuilding the damaged heart: Mesenchymal stem cells, cell-based therapy, and engineered heart tissue.. Physiol. Rev..

[21] Heldman A.W., DiFede D.L., Fishman J.E. (2014). Transendocardial mesenchymal stem cells and mononuclear bone marrow cells for ischemic cardiomyopathy: The TAC-HFT randomized trial.. JAMA.

[22] Bartunek J., Behfar A., Dolatabadi D. (2013). Cardiopoietic stem cell therapy in heart failure: The C-CURE (Cardiopoietic stem cell therapy in heart failure) multicenter randomized trial with lineage-specified biologics.. J. Am. Coll. Cardiol..

[23] Bolli R., Mitrani R.D., Hare J.M. (2021). A Phase II study of autologous mesenchymal stromal cells and c‐kit positive cardiac cells, alone or in combination, in patients with ischaemic heart failure: The CCTRN CONCERT‐HF trial.. Eur. J. Heart Fail..

[24] Choudhury T., Mozid A., Hamshere S. (2017). An exploratory randomized control study of combination cytokine and adult autologous bone marrow progenitor cell administration in patients with ischaemic cardiomyopathy: The REGENERATE‐IHD clinical trial.. Eur. J. Heart Fail..

[25] Qayyum A.A., van Klarenbosch B., Frljak S. (2023). Effect of allogeneic adipose tissue‐derived mesenchymal stromal cell treatment in chronic ischaemic heart failure with reduced ejection fraction – the science trial.. Eur. J. Heart Fail..

[26] Dib N., Dinsmore J., Lababidi Z. (2009). One-year follow-up of feasibility and safety of the first U.S., randomized, controlled study using 3-dimensional guided catheter-based delivery of autologous skeletal myoblasts for ischemic cardiomyopathy (CAuSMIC study).. JACC Cardiovasc. Interv..

[27] Henry T.D., Pepine C.J., Lambert C.R. (2017). The Athena trials: Autologous adipose‐derived regenerative cells for refractory chronic myocardial ischemia with left ventricular dysfunction.. Catheter. Cardiovasc. Interv..

[28] Mathiasen A.B., Qayyum A.A., Jørgensen E. (2020). Bone marrow‐derived mesenchymal stromal cell treatment in patients with ischaemic heart failure: Final 4‐year follow‐up of the MSC‐HF trial.. Eur. J. Heart Fail..

[29] Duckers H., Houtgraaf J., Hehrlein C. (2011). Final results of a phase IIa, randomised, open-label trial to evaluate the percutaneous intramyocardial transplantation of autologous skeletal myoblasts in congestive heart failure patients: The SEISMIC trial.. EuroIntervention.

[30] Pokushalov E., Romanov A., Chernyavsky A. (2010). Efficiency of intramyocardial injections of autologous bone marrow mononuclear cells in patients with ischemic heart failure: A randomized study.. J. Cardiovasc. Transl. Res..

[31] Perin E.C., Willerson J.T., Pepine C.J. (2012). Effect of transendocardial delivery of autologous bone marrow mononuclear cells on functional capacity, left ventricular function, and perfusion in chronic heart failure: The FOCUS-CCTRN trial.. JAMA.

[32] Santoso T., Siu C.W., Irawan C. (2014). Endomyocardial implantation of autologous bone marrow mononuclear cells in advanced ischemic heart failure: A randomized placebo-controlled trial (END-HF).. J. Cardiovasc. Transl. Res..

[33] Qayyum A.A., Mouridsen M., Nilsson B. (2023). Danish phase II trial using adipose tissue derived mesenchymal stromal cells for patients with ischaemic heart failure.. ESC Heart Fail..

[34] Teerlink J.R., Metra M., Filippatos G.S. (2017). Benefit of cardiopoietic mesenchymal stem cell therapy on left ventricular remodelling: Results from the Congestive Heart Failure Cardiopoietic Regenerative Therapy (CHART‐1) study.. Eur. J. Heart Fail..

[35] Perin E.C., Silva G.V., Zheng Y. (2012). Randomized, double-blind pilot study of transendocardial injection of autologous aldehyde dehydrogenase-bright stem cells in patients with ischemic heart failure.. Am. Heart J..

[36] Perin E.C., Sanz-Ruiz R., Sánchez P.L. (2014). Adipose-derived regenerative cells in patients with ischemic cardiomyopathy: The precise trial.. Am. Heart J..

[37] Perin E.C., Borow K.M., Silva G.V. (2015). A phase II dose-escalation study of allogeneic mesenchymal precursor cells in patients with ischemic or nonischemic heart failure.. Circ. Res..

[38] Perin E.C., Borow K.M., Henry T.D. (2023). Randomized trial of targeted transendocardial mesenchymal precursor cell therapy in patients with heart failure.. J. Am. Coll. Cardiol..

[39] Perin E.C., Silva G.V., Henry T.D. (2011). A randomized study of transendocardial injection of autologous bone marrow mononuclear cells and cell function analysis in ischemic heart failure (FOCUS-HF).. Am. Heart J..

[40] Patel A.N., Henry T.D., Quyyumi A.A. (2016). Ixmyelocel-T for patients with ischaemic heart failure: A prospective randomised double-blind trial.. Lancet.

[41] Williams A.R., Suncion V.Y., McCall F. (2013). Durable scar size reduction due to allogeneic mesenchymal stem cell therapy regulates whole-chamber remodeling.. J. Am. Heart Assoc..

[42] Tompkins B.A., Rieger A.C., Florea V. (2018). Comparison of mesenchymal stem cell efficacy in ischemic versus nonischemic dilated cardiomyopathy.. J. Am. Heart Assoc..

[43] Hare J.M., DiFede D.L., Rieger A.C. (2017). Randomized comparison of allogeneic versus autologous mesenchymal stem cells for nonischemic dilated cardiomyopathy.. J. Am. Coll. Cardiol..

[44] Jeevanantham V., Butler M., Saad A., Abdel-Latif A., Zuba-Surma E.K., Dawn B. (2012). Adult bone marrow cell therapy improves survival and induces long-term improvement in cardiac parameters: A systematic review and meta-analysis.. Circulation.

[45] Kandala J., Upadhyay G.A., Pokushalov E., Wu S., Drachman D.E., Singh J.P. (2013). Meta-analysis of stem cell therapy in chronic ischemic cardiomyopathy.. Am. J. Cardiol..

[46] Varzideh F., Gambardella J., Kansakar U., Jankauskas S.S., Santulli G. (2023). Molecular mechanisms underlying pluripotency and self-renewal of embryonic stem cells.. Int. J. Mol. Sci..

[47] van der Spoel T.I.G., Gathier W.A., Koudstaal S. (2015). Autologous mesenchymal stem cells show more benefit on systolic function compared to bone marrow mononuclear cells in a porcine model of chronic myocardial infarction.. J. Cardiovasc. Transl. Res..

[48] Shen T., Xia L., Dong W. (2021). A systematic review and meta-analysis: Safety and efficacy of mesenchymal stem cells therapy for heart failure.. Curr. Stem Cell Res. Ther..

[49] Li X., Hacker M. (2017). Molecular imaging in stem cell-based therapies of cardiac diseases.. Adv. Drug Deliv. Rev..

[50] Jeevanantham V., Butler M., Saad A., Abdel-Latif A., Zuba-Surma E.K., Dawn B. (2012). Adult bone marrow cell therapy improves survival and induces long-term improvement in cardiac parameters/clinical perspective.. Circulation.

[51] van der Spoel TIG, Jansen of Lorkeers SJ, Agostoni P. (2011). Human relevance of pre-clinical studies in stem cell therapy: Systematic review and meta-analysis of large animal models of ischaemic heart disease.. Cardiovasc. Res..

[52] Mushtaq M., DiFede D.L., Golpanian S. (2014). Rationale and design of the Percutaneous Stem Cell Injection Delivery Effects on Neomyogenesis in Dilated Cardiomyopathy (the POSEIDON-DCM study): A phase I/II, randomized pilot study of the comparative safety and efficacy of transendocardial injection of autologous mesenchymal stem cell vs. allogeneic mesenchymal stem cells in patients with non-ischemic dilated cardiomyopathy.. J. Cardiovasc. Transl. Res..

